# Real-World Application of Insulin Pump Therapy Among Patients With Type 1 Diabetes in China: A Cross-Sectional Study

**DOI:** 10.3389/fendo.2022.891718

**Published:** 2022-06-10

**Authors:** Lili Huo, Wei Deng, Ling Lan, Wei Li, Jonathan E. Shaw, Dianna J. Magliano, Linong Ji

**Affiliations:** ^1^ Department of Endocrinology, Beijing Jishuitan Hospital, Beijing, China; ^2^ Department of Clinical Diabetes and Epidemiology, Baker Heart and Diabetes Institute, Melbourne, VIC, Australia; ^3^ Department of Epidemiology and Preventive Medicine, Monash University, Melbourne, VIC, Australia; ^4^ Department of Endocrinology and Metabolism, Peking University People’s Hospital, Beijing, China

**Keywords:** type 1 diabetes, insulin pump therapy, China, hypoglycaemia, blood glucose control

## Abstract

**Background:**

Although insulin pump therapy is an important treatment modality for patients with type 1 diabetes, rates of pump use appear to vary broadly internationally. This study aimed to investigate the application of insulin pump therapy among patients with type 1 diabetes in China.

**Methods:**

Data were collected from the Type 1 Diabetes Mellitus in China: Coverage, Costs and Care Study (3C Study). A total of 779 participants from this cross-sectional study were included. Multivariable logistic regression was used for data analysis.

**Results:**

The median (interquartile range) age at diagnosis of diabetes was 17 (10–28) years and the duration of diabetes was 4 (1–8) years. Among 779 patients, only 89 patients (11.4%) used an insulin pump to control blood glucose. A statistically significant difference was found in HbA1c favoring insulin pump therapy (8.3 ± 1.7% vs. 9.2 ± 2.6%) without obvious differences for severe hypoglycaemia. There were higher proportions of patients with no smoking, frequent daily intake of fruits and vegetables, and adequate self-blood glucose monitoring among patients with insulin pump therapy as compared to those using multiple daily insulin injections. Logistic regression analysis showed that younger age at diagnosis, longer duration of diabetes, higher education level of family members, and higher household income were associated with the use of an insulin pump.

**Conclusions:**

Data from 3C Study demonstrated that only a minority of patients with type 1 diabetes in China utilize insulin pump therapy. Insulin pump therapy was associated with better blood glucose control and self-management. Patients with younger age at diagnosis and longer duration of diabetes, and patients with better socioeconomic status were more likely to use an insulin pump.

## Introduction

Type 1 diabetes is characterized by an autoimmune β-cell destruction that leads to absolute insulin deficiency and permanent reliance on exogenous insulin. Nowadays the use of intensified insulin therapy is the standard treatment in patients with type 1 diabetes from onset of the disease. Patients with type 1 diabetes are currently treated with either multiple daily insulin injections (MDI) or insulin pumps. In the last two decades, there has been an increasing number of patients using insulin pumps for the treatment of type 1 diabetes, owing to its ability to more closely simulate the physiologic pattern of insulin secretion. However, there is substantial variability among countries in the use of this treatment modality, even among developed Western countries. In light of the T1D Exchange registry, 64% of patients with type 1 diabetes in the United States use insulin pump therapy with an upward trend in pump use rate from 56% to 64% between 2011 and 2016 (1). Data on more than 54,000 young people with type 1 diabetes from three large registries showed that England and Wales had the lowest rate of insulin pump use of 14% as compared to 41% in Germany and Austria, and 47% in the United States (2). The uptake of insulin pump therapy varies widely possibly depending on the health care systems, insurance coverage and economic levels. Little is known about the application of insulin pumps among patients with type 1 diabetes in China. One recent survey from China reported the proportion of patients using insulin pumps to be as low as 15.21% among type 1 diabetes, which only included newly diagnosed children < 14 years of age (3).

Extensive research has been performed on the effects of insulin pumps on glycemic control and has established the efficacy and safety of pump use. A recent meta-analysis including 25 randomized controlled trials (RCTs) showed that pump therapy was associated with 0.37% lower HbA1c than MDI and a lower incidence of nocturnal hypoglycemia in patients with type 1 diabetes (4). The advantage of pump therapy over MDI have been demonstrated even when MDI includes a long acting insulin analog (glargine) (5, 6). Data from real world studies complement information reported in clinical trials. The pooled analysis of 3 large registries from the United States and Europe found a lower mean HbA1c of 0.5% among pump users versus MDI users (8.0 vs. 8.5%) (2). Translating the benefits of pump therapy into different clinical settings has been found to differ among centers and countries depending on insurance coverage and providers’ preference. The aim of the current study was to investigate the application of and barriers to insulin pump therapy among patients with type 1 diabetes in China, which may contribute to better understanding the existing gap of treatment selection.

## Methods

### Data Source and Study Design

Data were obtained from the Type 1 Diabetes Mellitus in China: Coverage, Costs, and Care Study (3C study), which was a cross-sectional study that aimed to investigate the coverage, cost and care of type 1 diabetes in 2 regions of China: Beijing and Shantou. The study included eight tertiary (six in Beijing and two in Shantou), five secondary (three in Beijing and two in Shantou) and six primary (four in Beijing and two in Shantou) health care facilities with active diabetes clinics, which were willing and able to participate in the study. The design of the 3C study has previously been described in detail (7). The study conducted a three-year retrospective review of hospital records to identify people with type 1 diabetes. People aged <6 months at the diagnosis of type 1 diabetes were excluded from this study. A total of 849 participants were sequentially enrolled. Face-to-face interviews were given to participants or their parents (if <15 years of age) by trained investigators during July/September 2011 and January/February 2012 using a pre-tested structured questionnaire. Participants’ anthropometrics were measured using standardized techniques. Waist circumference was measured through thin clothing using a non-stretchable fiber measuring tape. Blood pressure was measured twice with a mercury sphygmomanometer after sitting for at least 10 min, and was rounded to the nearest 2 mmHg. The average of the two readings was calculated. Venous blood samples were taken, and HbA1c and lipid profile were tested in local hospital laboratories. Seventy participants were excluded from our analysis due to missing data of HbA1c.

The diagnosis of type 1 diabetes developing after six months of age should be documented for all participants. Additionally, they met at least 1 of the followings: diagnosed ≤ 35 years of age, deficient C-peptide levels, signs of repeated ketosis if insulin discontinued for over one week during the first six months after diagnosis, ketoacidosis or uncontrolled hyperglycaemia if treated with oral antidiabetic drugs which resolved with insulin therapy. The assessment of diabetes self-care activities covered the previous 7 days (8). Diabetes diet was defined as that patient has an individualized food plan based on food preferences, schedule and physical activity (9). Controlled diet was defined as ≥ 5 days of meals in a week following the instruction on diabetes diet. Frequent intake of fruits and vegetables was defined as ≥ 5 days in a week following the recommendation on the intake of fruits and vegetables [five or more servings of fruits and vegetables per day. A serving of fruit and vegetables is equal to about 125ml in volume (10)]. The time of physical activity per week was categorized into ≥150 min and < 150 min. Non-smoker was defined as participants who were not current smokers or they had not smoked any amount of tobacco during the last year before enrolment. Self-blood glucose monitoring (SMBG) adherence was determined by the number of days of blood glucose tested in the previous week and was graded as adequate (≥5 days) and inadequate adherence (< 5 days). A severe hypoglycaemic event was characterized by altered mental and/or physical status requiring assistance for treatment of hypoglycemia in the past three months.

### Statistical Analysis

All data were entered into a database (EpiData version 3.1, EpiData Association, Odense, Denmark). Statistical analyses were performed using SPSS statistical software (version 22.0, SPSS Inc., Chicago, IL, USA). Data are presented as median (25th, 75th quartile), mean ± SD or n (%). Differences between two groups for normally distributed numeric variables were assessed using the two-sample t test. For comparison of numeric variables with skewed distributions, the Mann-Whitney U test was used. Assessment of differences in proportions between two groups was done by using a chi-square test. Multiple logistic regression was performed to identify the factors independently associated with the use of an insulin pump. Explanatory variables included in the model were gender, age (<20 years, 20-29 years, 30-39 years, ≥40 years), age at diagnosis (<10 years, 10-19 years, 20-29 years, ≥30 years), duration of diabetes, household income, highest education level of family members, location of residence, and insurance status. P-values<0.05 were considered statistically significant.

## Results

A total of 779 participants with type 1 diabetes were studied. Of these, 89 (11.4%) participants used an insulin pump. Demographic characteristics of the participants by insulin regimens are presented in **Table 1**. There were more females among patients using an insulin pump as compared with those using MDI. A significantly higher proportion of patients with higher household income, higher levels of family education, and living in an urban setting were on insulin pump treatment. There was no significant difference in age and insurance status between groups with an insulin pump and with MDI. **Table 2** summarizes participants’ clinical and lifestyle characteristics by insulin regimens. Patients with an insulin pump had earlier age at diagnosis of diabetes and longer duration of diabetes. There were significantly higher proportions of patients with no smoking, frequent daily intake of fruits and vegetables, and adequate SMBG in patients using insulin pump therapy as compared to those using MDI. In addition, the insulin pump therapy group also had higher proportions of patients with physical activity ≥150 min per week and controlled diet than did the MDI group, but the differences were not statistically significant.

**Table 1:** Demographic characteristics of people with type 1 diabetes by insulin regimen.

	Total n = 779	Continuous insulin pump therapy n = 89	Multiple daily insulin injections n = 690	P value
Gender, males, n (%)	382 (49.0)	34 (38.2)	348 (50.6)	0.032
Age (years)	24 (13, 36)	22 (13, 35)	24 (14, 36)	0.270
Household income (¥/month)	3000 (2000, 6000)	5000 (3000, 10000)	3000 (2000, 5000)	0.000
Highest education level of family members, n (%)				0.000
Less than bachelor’s degree	519 (67.4)	38 (42.7)	481 (70.6)	
Bachelor’s degree or more	251 (32.6)	51 (57.3)	200 (29.4)	
Location of residence, n (%)				0.000
Rural area	338 (44.3)	18 (21.2)	320 (47.2)
Urban area	425 (55.7)	67 (78.8)	358 (52.8)
Insurance status, n (%)				1.000
Without insurance	691 (88.7)	79 (88.8)	612 (88.7)
With insurance	88 (11.3)	10 (11.2)	78 (11.3)

**Table 2:** Clinical and lifestyle characteristics of people with type 1 diabetes by insulin regimen.

	Total n = 779	Continuous insulin pump therapy n = 89	Multiple daily insulin injections n = 690	P value
Age at diagnosis (years)	17 (10, 28)	12 (8, 21)	18 (10, 29)	0.000
Duration of diabetes (years)	4 (1, 8)	6 (2, 19)	3 (1, 7)	0.000
Waist circumference (cm)	71.8 ± 11.0	70.3 ± 10.5	72.0 ± 11.1	0.202
BMI (Kg/m^2^)	20.0 ± 3.6	20.1 ± 3.6	20.0 ± 3.6	0.930
SBP (mmHg)	113(103, 120)	113 (100, 120)	113 (103, 120)	0.670
DBP (mmHg)	73 (66, 80)	71 (65, 77)	73 (66, 80)	0.172
HbA1c (%)	9.1 ± 2.5	8.3 ± 1.7	9.2 ± 2.6	0.014^*^
LDL-C (mmol/L)	2.7 (2.1, 3.2)	2.7 (2.2, 3.5)	2.6 (2.1, 3.2)	0.439
Triglyceride (mmol/L)	0.9 (0.6, 1.4)	0.8 (0.6, 1.2)	0.9 (0.6, 1.5)	0.103
Hypertension, n (%)	111 (15.5)	12 (14.6)	99 (15.6)	1.000
Smoking, n (%)	114 (14.6)	5 (5.6)	109 (15.8)	0.010
Diet control status, n (%)				0.536
Controlled	553 (71.0)	66 (74.2)	487 (70.6)	
Uncontrolled	226 (29.0)	23 (25.8)	203 (29.4)	
Fruits and vegetables, n (%)				0.016
Frequent	456 (58.5)	63 (70.8)	393 (57.0)
Less frequent	323 (41.5)	26 (29.2)	297 (43.0)
Physical activity, n (%)				0.565
≥150 min/week	471 (60.5)	51 (57.3)	420 (60.9)
< 150 min/week	308 (39.5)	38 (42.7)	270 (39.1)
Self-blood glucose monitoring, n (%)				0.002
Adequate	297 (38.1)	48 (53.9)	249 (36.1)
Inadequate	482 (61.9)	41 (46.1)	441 (63.9)
Severe hypoglycemia				0.926
No. of events	189	18	171
Rate per 100 person-yr	98.8	81.8	101.0

^*^Adjusted for gender, age and duration of diabetes.

**Figure 1:**
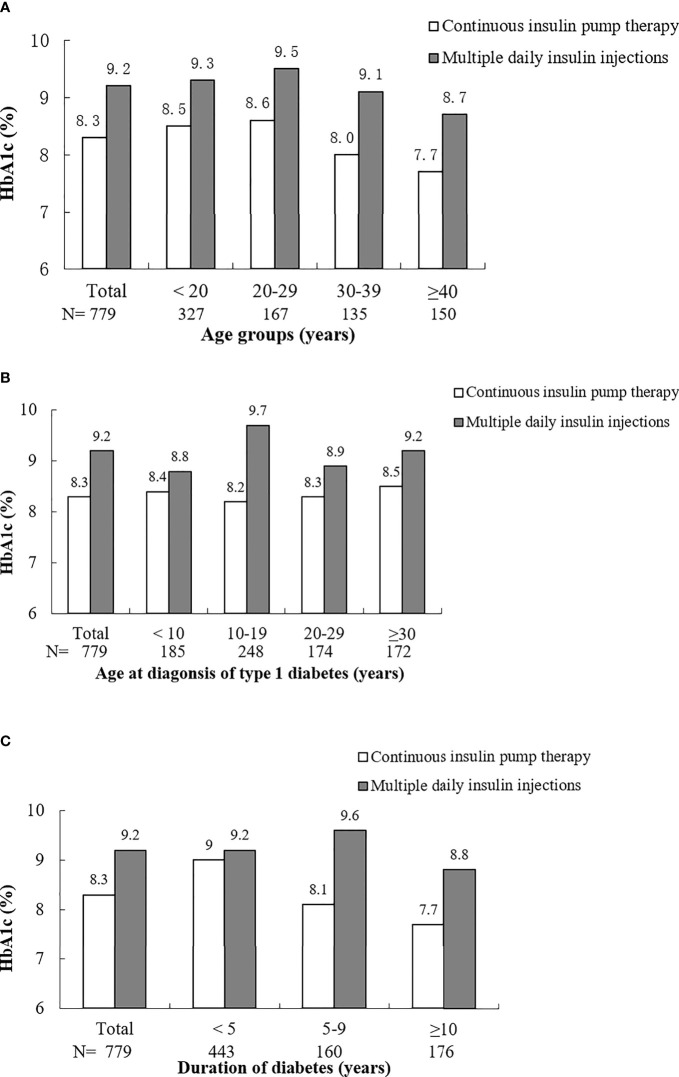
HbA1c of patients with continuous insulin pump therapy and multiple daily insulin injections by groups of age **(A)**, age at diagnosis of type 1 diabetes **(B)** and duration of diabetes **(C)**.

A lower average HbA1c level was observed among those with an insulin pump in contrast to those with MDI (8.3 ± 1.7% vs. 9.2 ± 2.6%). **Figure 1** shows that the HbA1c levels of insulin pump therapy group were consistently lower than those of the MDI group across different age groups, age at diagnosis groups and duration of diabetes groups. Insulin pump therapy seemed to achieve a lower HbA1c level among those diagnosed at the age of 10-19 years or with ≥5 years of duration of diabetes. In terms of rate of severe hypoglycaemia, no significant difference was found between groups with an insulin pump and with MDI.

**Figure 2:**
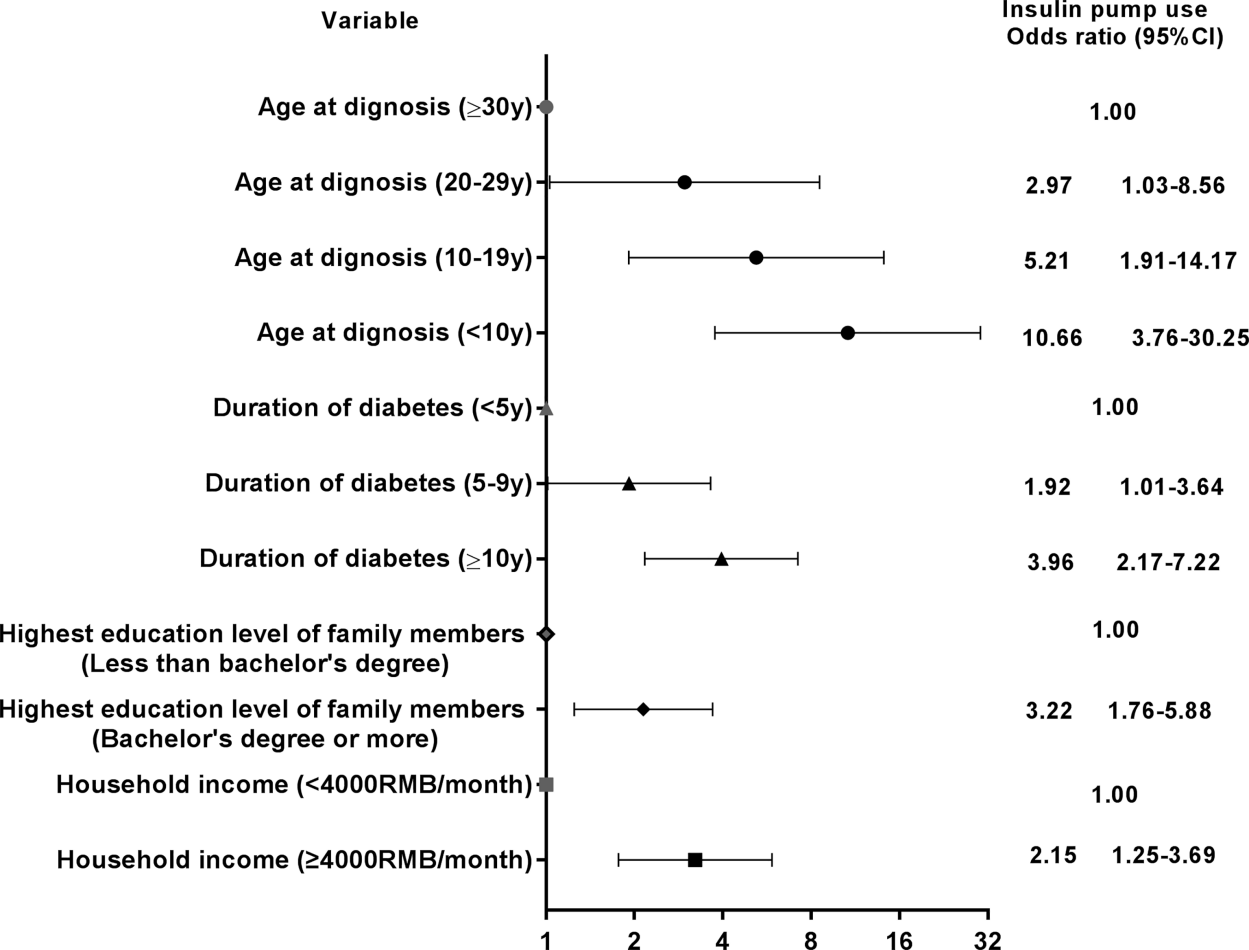
Adjusted odds ratios and 95% confidence interval (CI) between insulin pump use and different patient characteristics. Variables introduced in the multivariable analysis were age, sex, age at diagnosis, duration of diabetes, waist circumference, household income, location of residence, education level of family members and insurance status.

In logistic regression analysis, younger age at diagnosis of type 1 diabetes, longer duration of diabetes, higher education level of family members, and higher household income were associated with use of an insulin pump (**Figure 2**). Patients diagnosed with type 1 diabetes at age 20-29 years were nearly 3-fold more likely to use an insulin pump, 5.2-fold for patients diagnosed at age 10-19 years, and about 10-fold for patients diagnosed at age <10 years, as compared with those diagnosed at ≥30 years. Duration of diabetes 5-9 years and ≥10 years increased the likelihood of using an insulin pump by 92% and threefold, respectively, compared to a duration of diabetes <5 years. Higher education level was associated with 3.2-fold of likelihood of using an insulin pump compared to lower education level. Patients with higher household income were over twice as likely to use an insulin pump as were patients with lower household income.

## Discussion

This is one of very few studies to describe the application of insulin pump therapy among type 1 diabetes in clinical practice in China. Although in our previous study, utilization of an insulin pump has been reported to reduce the risk of poor glycemic control by 58% (11), the present analysis has allowed a more detailed investigation of the use of pump technology than previous work. In this cross-sectional study, only 11.4% of people with type 1 diabetes used an insulin pump. Use of insulin pump treatment was associated with better glycemic control than MDI with a mean HbA1c difference of 0.9%, but no difference in the rate of severe hypoglycemic events. This difference in HbA1c was greater than was found by RCTs (4), which is likely related to differences between pump users and MDI users. It may be assumed that patients accepting and taking advantage of insulin pumps represent a group with specific clinical and behavioral characteristics. In our study, there were more females among insulin pump users. This is consistent with a Swedish study based on data from hospital outpatient clinics (12), which might be attributable to the different attitudes about treatment modalities by sex. We also found that there were significantly higher proportions of patients with no smoking, frequent daily intake of fruits and vegetables, and adequate SMBG in insulin pump users. It is possible that individuals who use pump therapy are more highly motivated to control blood glucose with healthy behaviors and have better adherence to the prescribed medical regimen and intensified glucose monitoring. The benefits of healthy diet and SMBG on glycemic control have been observed in many studies (11, 13, 14).

Of note, no difference in the rate of severe hypoglycemic events between insulin pump users and MDI users was found in our study. In terms of hypoglycemia, RCTs and meta-analyses of RCTs reported inconclusive results. A Cochrane 2010 meta-analysis included 23 reported RCTs reported no difference in the rate of non-severe hypoglycemic events, but reduced severe hypoglycemia with pump therapy (15). Another recent meta-analysis including 25 RCTs found no difference in minor or severe hypoglycemic events, but a lower incidence of nocturnal hypoglycemia was observed in the pump therapy group (4). A recently published longitudinal study using data from the Western Australian Children’s Diabetes Database found that the pump cohort had more than double the rate of severe hypoglycemia events than the MDI cohort in the year prior to starting pump therapy (16). After a mean follow-up of 4 years the rate of severe hypoglycemia in the pump cohort decreased by more than 50%, to a rate similar to that of the MDI cohort (16). This is consistent with our results. When insulin pumps were first used, a history of severe hypoglycemia was a key determining factor for commencement of pump therapy. In our study, pump users had an earlier age of diagnosis and longer duration of diabetes, which may indicate less residual endogenous insulin secretion and higher risk of hypoglycemia. Therefore, caution should be taken when interpreting hypoglycemia, which is likely related to differences between pump users and MDI users rather than the insulin delivery modality.

In logistic regression analysis, younger age at diagnosis of type 1 diabetes, longer duration of diabetes, higher education level of family members, and higher household income were associated with use of an insulin pump to control blood glucose. Education level and household income are often inter-related and represent socioeconomic status. As insulin pump therapy is more expensive than MDI and currently there is no reimbursement for insulin pumps and related supplies in China, the selection of an insulin pump could be influenced by social factors, which may lead to health inequalities. Advocacy efforts should be directed to people of lower socioeconomic status who do not typically have chance to benefit from advanced technology. In addition, in light of the young age of this population, higher education level of family members can help a patient with type 1 diabetes have more confidence to properly use the insulin pump and cope with the technological demands. Although there is no overall consensus on indications for insulin pump therapy, it is widely accepted that those with frequent, severe hypoglycemia and/or hypoglycemia unawareness may derive particular benefit (17). Insulin pump therapy is routinely started at the time of diagnosis in some countries. UK guidance from the National Institute for Health Care and Excellence (NICE) has specified that pumps should be considered for children <12 years of age and for those aged >12 years, the use of pump therapy is only recommended if an individual has disabling hypoglycaemia or poorly controlled diabetes (HbA1c >8.5%) on MDI (18). The newly-revised edition of Chinese Insulin Pump Clinical Guideline (2021) has listed type 1 diabetes as an indication with no special recommendation or restriction by age or glycaemic control (19). In our study we found that pump use increased with a younger age of diagnosis, which could reflect the expansion of this technology in pediatric practice.

In this study, we present a snapshot of the real-life use of insulin pump among people with type 1 diabetes with a relatively large sample size that was recruited from primary, secondary and tertiary levels of health facilities. Several limitations should be considered when interpreting the results. First, this survey was conducted nearly 10 years ago when short and long-acting insulin analogs were not widely used in China, which may amplify the differences between the pump therapy and MDI. Additionally, ten years is a long time when referred to the adoption of innovative technology, which has disabled our analysis from reflecting the current revolution in insulin pumps. However, a study published in 2017 using data from Guangdong Type 1 Diabetes Mellitus Translational Medicine Study, which was conducted in 16 centers in Guangdong province between 2011 and 2014, reported 12.3% of rates of pump use (20). Another survey that was conducted in Chinese children with type 1 diabetes between 2012 and 2015 from 25 major cities reported 15.21% of patients receiving insulin pump therapy (3). The data in our study showed that the proportion of patients using pump therapy was 17.2% among those with age of diagnosis < 14 years, and 10.2% among those diagnosed < 14 years of age and with < 1 year of duration of diabetes. Therefore, our data on the rate of pump use are comparable to those from recent study or survey, which indicates that the situation of application of insulin pump therapy hasn’t changed much in China. Second, the duration of pump use was not collected, which meant that we could not evaluate the sustainability of the glycaemic benefit of insulin pump therapy. Third, the usage of continuous glucose monitors (CGM) was not recorded in this population. The advantages of CGM use for HbA1c and hypoglycaemia reduction have been shown both in trials (21, 22) and registry data (23). CGM is not publicly funded and the clinical application of CGM was very limited during the study period in China, therefore, the contribution of CGM might not affect the overall findings. Lastly, only severe hypoglycemia was reported and there were no data on classification of hypoglycemia by time of day for this population.

In summary, our study shows that only a minority of patients with type 1 diabetes in China utilize insulin pump therapy, which is associated with better blood glucose control and self-management. Patients with younger age at diagnosis and longer duration of diabetes, and patients with better socioeconomic status were more likely to use an insulin pump. The current study helps to obtain a comprehensive picture of the availability of insulin pumps among type 1 diabetes in clinical practice in China. In the last few years, as pump technology has been developed rapidly, it will be even more important to ensure that our socially disadvantaged patients also have access to such treatment advancement. Efforts to identify the barriers of inequalities in treatment selection are needed.

## Data Availability Statement

The raw data supporting the conclusions of this article will be made available by the authors, without undue reservation.

## Ethics Statement

The studies involving human participants were reviewed and approved by the Ethics Committees of the Beijing Children’s Hospital, Peking University Health Science Centre, the First Affiliated Hospital of Shantou University Medical College and the Second Affiliated Hospital of Shantou University Medical College. Written informed consent to participate in this study was provided by the participants’ legal guardian/next of kin.

## Author Contributions

LH performed data analysis and interpretation, and wrote the first draft of the manuscript; LJ conceived the study, made substantial contributions to data interpretation, and reviewed and revised the manuscript; WD, LL, and WL assisted in data preparation and analysis, and reviewed and revised the manuscript; JS and DM made a substantial contribution to data analysis and interpretation, and reviewed and revised the manuscript. All authors approved the final version of the article. LJ is the guarantor of this work and, as such, has full access to all of the study data and takes responsibility for data integrity and the accuracy of data analysis.

## Funding

This study was supported by Beijing Jishuitan Hospital Elite Young Scholar Programme (No. XKGG202118). This study received funding from Sanofi Diabetes. The funders had no role in study design, data collection and analysis, decision to publish, or preparation of the manuscript.

## Conflict of Interest 

JS has received funding from Novo Nordisk, Eli Lilly and Sanofi.

The remaining authors declare that the research was conducted in the absence of any commercial or financial relationships that could be construed as a potential conflict of interest.

## Publisher’s Note

All claims expressed in this article are solely those of the authors and do not necessarily represent those of their affiliated organizations, or those of the publisher, the editors and the reviewers. Any product that may be evaluated in this article, or claim that may be made by its manufacturer, is not guaranteed or endorsed by the publisher.
